# Warmforming Flow Pressing Characteristics of Continuous Fibre Reinforced Thermoplastic Composites

**DOI:** 10.3390/polym14225039

**Published:** 2022-11-21

**Authors:** Benjamin Gröger, David Römisch, Martin Kraus, Juliane Troschitz, René Füßel, Marion Merklein, Maik Gude

**Affiliations:** 1 Institute of Lightweight Engineering and Polymer Technology, Technische Universität Dresden, Holbeinstraße 3, 01307 Dresden, Germany; 2 Institute of Manufacturing Technology, Friedrich-Alexander-Universität Erlangen-Nürnberg, Egerlandstraße 13, 91058 Erlangen, Germany

**Keywords:** thermoplastic composite, clinching, joining, material behaviour, FEM, compaction

## Abstract

The paper presents research regarding a thermally supported multi-material clinching process (hotclinching) for metal and thermoplastic composite (TPC) sheets: an experimental approach to investigate the flow pressing phenomena during joining. Therefore, an experimental setup is developed to compress the TPC-specimens in out-of-plane direction with different initial TPC thicknesses and varying temperature levels. The deformed specimens are analyzed with computed tomography to investigate the resultant inner material structure at different compaction levels. The results are compared in terms of force-compaction-curves and occurring phenomena during compaction. The change of the material structure is characterized by sliding phenomena and crack initiation and growth.

## 1. Introduction

**Figure 1:**
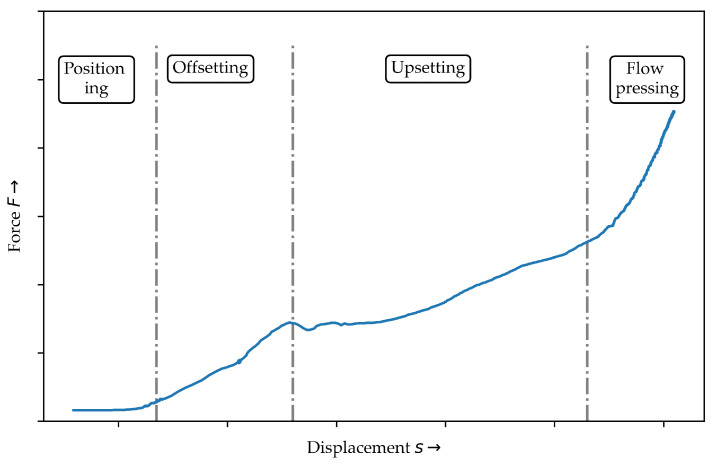
Measured force-displacement curve of the hotclinching process and the four phases of clinching based on [11].

The demand for lightweight structures especially in automotive application increases with the requirement of reducing global ${\mathrm{CO}}_{2}$—emission. An appropriate way is the method of multi-material-design. This enhancement for body-in-white structures leads to a mass reduction, which decreases the fuel emission. With respect to the limitation of global resources, the decomposition of multi-material-assemblies has to be considered. For this lightweight purpose, composites with high specific mechanical properties are used [1]. Additionally, the controllable ductility at elevated temperatures allows joining of the thermoplastic composites (TPC) lightweight structures to metal using novel joining technologies [2,3,4,5]. With the focus on recyclable joints and sustainability, clinching as a cost efficient mechanical joining technology seems appropriate [6,7]. It is widely used in industrial applications [8] and a further development enables the joining of less ductile materials [9] or thick sheets [10]. Furthermore, the clinching technologies for composites of glass-fibre-reinforced (GFRP) thermosetting polymers [11], carbon-fibre-reinforced (CFRP) thermosetting polymers [12,13,14], GFRP thermoplastic [15] (punch-sided) [16], (die-sided) or CFRP thermoplastic [17] are possible. In [3] a classification about different clinching processes for continuous fibre-reinforced thermoplastics is given. In this classification the complexity of clinching tools could be seen. Another differentiating feature is the given thermal support before or during the clinching process. In [18] the results of a thermally supported clinching process in comparison to a clinching process under room temperature shows a significant increase of the shear strength caused by less fibre failure in the neck area induced by the deformation process. In contrast, in the bottom area and the ring groove fibre fragments can be seen (cf. [19]). For a more detailed description of a clinching process four main phases are defined in [10] for metals and in [11] for composites. The four phases positioning, offsetting, upsetting and flow pressing can be seen in Figure 1. The offsetting phase is characterised by bending, fibre failure and reorientation around the punch and the area between the anvil and the punch. During the upsetting and flow pressing phase the undercut is formed. In these phases also the resultant material structure with the fibre fragment caused by the displacement in radial direction occurs. The compaction within plastic deformations and the flow pressing process are challenging phenomena in numerical simulations.

In [20], process phenomena for unidirectional (UD) carbon fibre (CF)/ polyetheretherketone (PEEK) with focus on transverse squeeze flow above melting temperature of the matrix are investigated. The specimens’ heights vary between one ply ( 0.125 mm) and eight plies ( 1.0 mm). The specimens were pressed between two heated plates and the changing of the dimension was measured. It could be seen that the change of the width dimensions (transverse to the fibre direction) as a result of the applied normal pressure is the dominant phenomenon. Further investigations of squeeze flow phenomena are performed by [21] for consolidated specimens, squeeze flows during sheet moulding compounds [22] and summarized in [23]. So the width was correlated with the nominal pressure. Furthermore, from the resultant graph and the observed material structure, four phases are inferred. Moreover, “fibre jetting” in the middle of the specimen with the highest applied pressure is observed. Investigations for compaction of fibres with thermosetting matrix are performed by [24]. The authors presented a newly developed test rig with just one load level. The results are promising but just offer the evaluation of the material structure after a complete curing cycle. Investigations of compaction behaviour for UD fibres, the resultant inner material structure and the resultant mechanical properties are given in [25]. The compaction stress of 21 MPa leads to a compaction without damage or flow pressing phase. In [26], boundary integral methods with the squeeze flow of resin and UD– fibres on micro scale are investigated. The theoretical results of fibre-reinforced specimen were compared to the squeeze flow of pure resin. The results show a significant influence of the fibres to the flow front. A macroscopic view on this phenomena is perormed by [27] for modeling the flow of an UD-tape between two flat plates. A test rig with a closed cavity is used by [28] for a 2-dimensional (2D) modeling approach for a flow front by randomly distributed fibres tapes on one half of the cavity. Ref. [29] also used a closed cavity to develop a 3-dimensional (3D) modeling approach for compaction of CF/polyetherketoneketone (PEKK) platelets and methods of computed tomography (CT) and micographs to investigate the resultant meso structure. Therefore, the compaction is analysed in terms of the resultant fibre orientation at the different compaction levels and phenomenological description of the compaction behaviour.

In [30], the compaction behavior above melting temperature of CF/PEEK and CF/ polyphenylensulfide (PPS) at forming processes is investigated by numerical simulation and an experimental test setup. It could be shown that squeeze flows and fibre bleeding, and transverse winding in the outer area occur. A numerical description is made for the prediction of the final compaction thickness under a given load. A simulation approach of the squeeze flow show the flow velocities in both in-plane and out-of-plane direction [31].

Due to the high compression forces during compaction, the tool-ply interaction as one of the forming phenomena has to be taken into account [32]. This interaction in terms of friction depends on the state of the matrix and is often described by the Stribeck curve with the three different friction types [33]. At a solid matrix state, the two contact partners slide along each other with a relatively high friction coefficient (FC). With increasing speed, pressure and temperature a mixed lubrication friction type with decreasing FC results. When applying process temperatures above melting temperature of the matrix a fluid film between fibres and metal sheet leads to lubrication and therefore to a hydrodynamic friction. The friction between tool and composite ply is often investigated by pull-out test [33,34,35], ABlock-on-Ring tribometer [36], based on rheological test setups [37] but also novel testing methods can be used [38].

Nevertheless, it can be seen in the literature, there is a lack of information for global unidirectional distributed fibres especially for high compaction levels occuring during clinching above room temperature next to the Vicat-temperature of the thermoplastic matrix. The present paper investigates this gap to enable both a better phenomenological understanding based on the CT-scans and in combination with the force-compaction-graphs an accurate material modeling to simulate the joining process. Therefore, the tool-ply behaviour in terms of friction is investigated with a pin-on-disk-test. Therefore, the experimental setup, the used material and the properties of CT-scans at different load stages are described in detail. For a wide range of applications, various sheet thicknesses are investigated.

## 2. Clinching of Fibre-Reinforced Plastics

**Figure 2:**
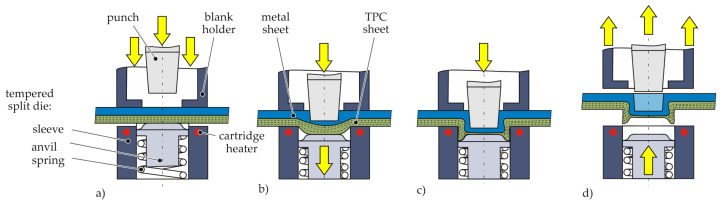
Process states of the thermally assisted clinching process [3]; (**a**) Positioning (**b**) Offsetting (**c**) Upsetting and Flow Pressing (**d**) Finished joint.

**Figure 3:**
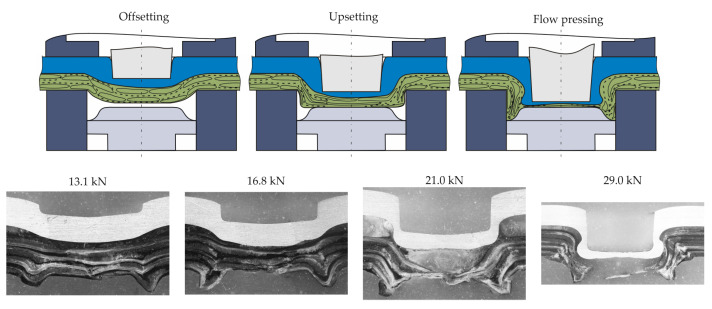
Schematic illustration and micrographs of the material structure at the different phases.

The present investigations are based on a thermal supported clinching process called “hotclinching” developed by [18] and is illustrated in Figure 2. The process is a one step process which requires neither any auxiliary parts nor preliminary steps. The special feature is a tool concept with a tempered split die. The die consists of a rigid tempered sleeve and a spring-loaded anvil. In [18], different temperature levels and the resultant material structure are investigated. Therefore, the thermally assistance leads to a more ductile behaviour of the TPC and reduces the damage in the joining zone, especially in the punch feed and neck area, where fibre bending occurs. In the bottom area, fibre failure are caused by tension in fibre direction and pressure in thickness direction (cf. Figure 3— 13.1 kN). It could be shown, that joining process temperatures next to Vicat temperature lead to higher tensile strength and a joint with sufficient undercut, neck thickness and bottom thickness. Heating above melting temperature on the other hand leads to insufficient undercuts. For a deeper knowledge, in [19], the clinching joint for aluminium-multi-layered unidirectional glass fibre (GF)/polyamide 6 (PA6) sheet is investigated by CT. Since the process temperature is below the melting point, it could be proven that no independent matrix flow processes occur. Additionally, ref. [3] shows a detailed view of the tools and resultant material structure and compares this clinching process with other common clinching technologies. The processes with pre-holes and without pre-holes are also taken into account as thermally assisted joining technologies [3]. The absence of preliminary steps, e.g., drilling pre-holes, is a benefit due to the less required positioning accuracy for joints with pre-holes.

Clinching at room temperature leads to a failure-dominated material structure (matrix, fibre and interfibre failure). In comparison to joining technologies with pre-holes at room temperature no delamination (cf. [17]) and a changed load bearing behaviour due to the resultant material structure occur. In [39] a comparison of drilled and pierced holes are made. For tensile tests, the stiffness of both are similar whereas the failure strain for the pierced specimens are higher.

The four phases of the one-step process are illustrated in Figure 2. At first, the metal and composite sheet were positioned and clamped by the sleeve, anvil and the blank holder (cf. Figure 2a). At the beginning, the initial anvil position is above the sleeve and generates hydrostatic pressure. After warming up below melting temperature the punch moves downwards and the offsetting begins (cf. Figure 2b).

When the anvil reaches a mechanical stop, the third and fourth phase of upsetting and flow pressing occurs (cf. Figure 2c). After the punch reached the end position, the joining process is finished and the assembly can be released (cf. Figure 2d).

The formation of the undercut and the neck thickness occurs in the upsetting and flow pressing phase. Therefore, Figure 3 illustrates the phases (a) offsetting, (b) upsetting and (c) flow pressing in accordance to [11]. In addition, micrographs of these phases were added to give a better understanding of the formed material structure. It can be seen that the bottom thickness of the TPC decreases with increasing punch motion. For the longitudinal yarns, fibre failure occurs after a reorientation in the bending area.

**Figure 4:**
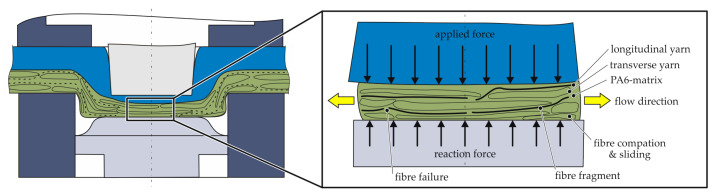
Schematic illustration of the occurring phenomena during upsetting phase as basis for the test rig development.

In the centre area under the punch (bottom area) fibre failure increases. The fragments were displaced in radial direction to the ring groove. The cross section of yarns in transverse direction changes during these compaction and displacement processes and the fibres are also displaced in radial direction. The phenomena are simplified and illustrated in Figure 4. In cause of the fibre failure in the bottom area it can be assumed, that in both fabric directions the compaction and squeeze flow phenomena are the major effects. For a deeper understanding of the clinching process it is appropriate to decompose the phenomena into main features. This enables a further development for a numerical modelling strategy and an optimized process. Therefore, the major effect of the compaction is the squeeze flow, which is investigated in a simplified test rig.

## 3. Materials and Methods

### 3.1. Material Specification

For the investigations an UD GF/PA6-sheet with three different thicknesses *d* is used. The material TEPEX^®^ dynalite 102-RGUDm317 with a measured fibre volume content of 44% is manufactured by Lanxess Bond Laminate. The geometric specifications are given in Table 1.

**Figure 5:**
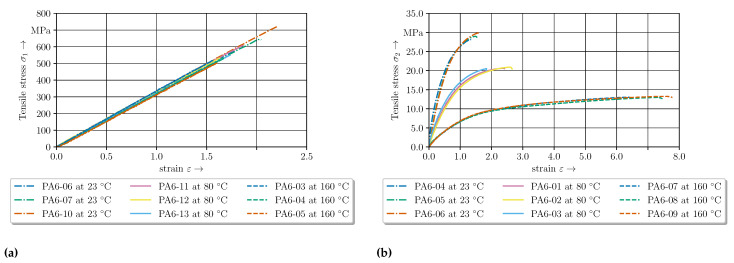
Experimental temperature-dependent stress strain curves for the three different temperature levels. (**a**) Experimental data for 0°-direction. (**b**) Experimental data for 90°-direction.

In Figure 5 the temperature and direction dependent material behaviour are shown. A detailed analysis is performed by [40]. Due to the UD layers a linear elastic behaviour with temperature dependent failure strain in fibre direction (0°) can be seen (Figure 5a). Transverse to fibre direction (90°), the in-plane tensile tests show a strong dependency of the initial stiffness and the ductile behaviour on the temperature (Figure 5b). Due to the matrix dominated material behaviour in transverse direction, a ductile behaviour can be assumed.

### 3.2. Experimental Setup and Characteristic Dimensions

#### 3.2.1. Flat Crush Test with Elevated Temperatures

**Figure 6:**
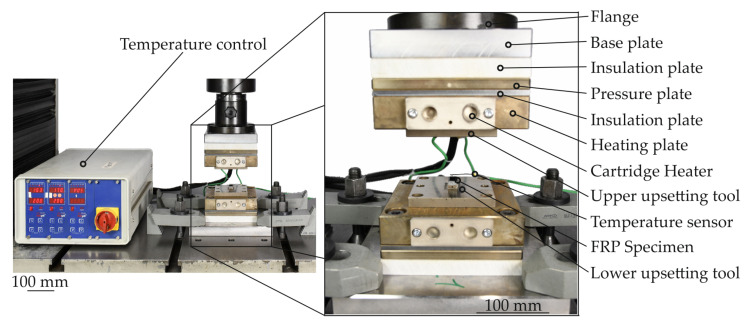
Experimental setup of the flat crush test.

For the flat crush test, which was carried out for the TPC specimens at different temperatures, a special hot flat crush tool was constructed (Figure 6). The tool enables both the upper and lower tools to be heated to the target temperatures using two cartridge heaters respectively. Two separate control circuits were used to keep the upper and lower upsetting tool at the target temperature by means of a temperature control unit via a PID system using one temperature sensor in each tool. To ensure that the heat introduced into the system via the cartridge heater is not conducted into the machine frame, several insulation plates were incorporated into the tool design to avoid excessive heat loss. In order to be able to make a representative assessment of the degrees of compaction being investigated and to ensure that the testing machine moves the defined distance for the desired compaction based on the initial sample height, a compensation curve for the tool was recorded. For this purpose, the upper and lower tool is brought into contact without the test specimen present and is subsequently subjected to a load while the elastic compensation curve is recorded. In this way, the elasticity of the tool and the testing machine can be compensated during testing. The tool itself was installed in a conventional universal testing machine of the type Walter and Bai 300 with a maximum force of 300 kN. The experimental setup with all the relevant components is shown in Figure 6.

**Figure 7:**
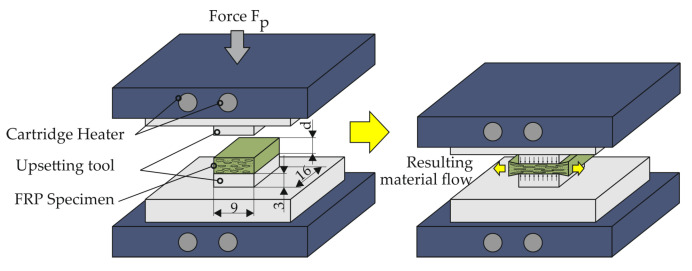
Schematic illustration of the process sequence of the plane-strain upsetting test.

In Figure 7 a schematic illustration of the flat crush test procedure and the dimensions of the upsetting tools and the test specimens is shown. Thereby, the upsetting tool had a width of 9 mm and a length of 16 mm. The test specimens made of GF/PA6 sheet were also cut to these dimensions. In order to test the conditions of the clinching process of 180 °C the compaction investigations are also performed below the melting temperature at three different temperature levels T of 160 °C, 180 °C and 200 °C. The chosen temperature levels are below the melting temperature of PA6. Only the 200 °C temperature level is around the Vicat temperature of PA6. In conclusion, no independent matrix flow process can occur (cf. [41]). At the given temperatures the matrix is more ductile in the solid state and the squeeze flow in transverse direction to the fibres can be described as a yielding. With respect to the technical relevance, for detailed investigations six different compaction levels are chosen focused on specimens with 2 mm thickness. The specimens with 1 mm and 4 mm thickness are compacted at three compaction levels. For every compaction level at a given temperature a novel specimen is used and evaluated. The test program is given in Table 2.

In the first step of the test procedure the initial thickness of the specimen is measured and transferred to the test software to calculate the individual compaction level. After positioning, the specimen is preloaded with 500 N to generate a full contact between the heated tools and the composite. Furthermore, warping or manufacturing imperfections at the surface of the TPC from cutting with a watercooled Axitom are eliminated. The heating process takes 30 s for the 1 mm and 60 s for the 2 mm specimen. The heating of the 4 mm specimens is set to 90 s to guarantee an isothermal temperature level. These heating times were evaluated by preliminary test with thermoelements. The compaction velocity of the punch after heating is defined with 1 mm/min. During the compaction process, the temperature is kept constant.

#### 3.2.2. Tribological Analysis with Modified Pin-on-Disk Test

**Figure 8:**
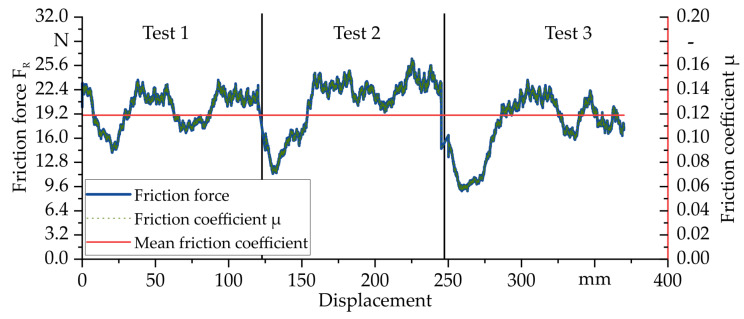
Diagram of the friction force/displacement curve and the corresponding and mean friction coefficient μ at 160 °C for 1.2343 and GF/PA6.

To characterise the friction conditions between the test specimens and the upsetting tool during the compaction tests, a modified pin-on-disk test at 160 °C was carried out. The test setup consists of a heated metal plate, which is heated by two ceramic heating elements. In addition, a friction tool with a friction surface of 5×6 ${\mathrm{mm}}^{2}$ made of 1.2343 with a polished surface, ensuring the same friction conditions as the upsetting tool, was mounted in a heated frame attached to a KUKA KR200-3 industrial robot. In this way, both the tool and the test specimen can be heated separately. The Kuka robot itself finally applies the defined force and sliding velocity. Before the tests, the transmitted normal force $F_{N}$ was set to 160 N via a load cell, resulting in a surface pressure of 5.37 MPa for the friction surface mentioned. During the test, the friction force $F_{R}$ is recorded via a 1 kN load cell mounted on the front panel of the friction test rig. The friction coefficient μ can be determined via Coulomb’s law

(1) $$F_{R}=\mu \;\cdot \;F_{N}\;,$$

using the previously determined normal force $F_{N}$ with which the friction tool is pressed onto the sample. Before carrying out the test, the contact surface was calibrated using pressure foil (Fujifilm prescale) to ensure an even distribution of pressure. The GF/PA6 sample with dimensions of 30 × 200 mm^2^ was finally placed on the preheated holder and preheated. The temperature of the sample was measured continuously. After the target temperature was reached, the process was started by first placing the tool on the sample with the defined normal force $F_{N}$. The tool was then moved in a defined path with a length of 125 mm and a speed of v= 100 mm/min at 160 °C over the surface of the sample. This procedure was repeated three times in order to determine an average value of the friction coefficient from the tests. The results of the friction characterization for the tool steel 1.2343 and GF/PA6 at 160 °C are shown in Figure 8. A mean coefficient of friction μ of 0.12±0.02 could be experimentally determined for this material combination.

### 3.3. Friction Compensation

According to Siebel’s compression force formula [42], the frictional force share of the forming force increases along with decreasing specimen thickness. Therefore, to ensure comparability of the stress-strain curves at different degrees of compaction, the curves were compensated with regard to the friction conditions present. For this purpose, reference was made to the work of Kappelner et al. [43], who investigated the determination of flow curves with the warm flat crush test. For this compensation, Equation (2)

(2) $$k_{f}=\frac{k_{w}}{\frac{exp(\frac{\mu \;\cdot \;l}{h})-1}{\frac{\mu \;\cdot \;l}{h}}+\frac{h}{4l}}\;,$$

was used according to [43], where $k_{f}$ is the compensated stress, $k_{w}$ is the uncompensated stress, μ is the coefficient of friction, for which a value of 0.12 has been determined experimentally using the modified pin-on-disk test. Furthermore, *h* is the initial sample height and *l* is the upsetting tool width of 9 mm. In this way, the stress in the component due to friction can be excluded, which has a great influence on the existing stress conditions and is, as can be seen from Equation (2), strongly dependent on the residual height of the sample.

### 3.4. Evaluation Methods

The compacted specimens were investigated by ex-situ CT. This non-destructive imaging method enables a detailed view of the inner material structure [44,45]. Therefore, different sections and views can be generated for the analysis with one specimen. This reduces the experimental effort by using one specimen at one compaction level instead of one specimen per section. The CT analysis are performed by a Phoenix X-ray Vtomex L450 with a 300 kV micro-focus X-ray tube. The specifications of the scans are listed in Table 3.

## 4. Results

The experimental results are presented in terms of compensated stress-compaction strain-curves and ex-situ CT analysis at specific compaction levels. At first, the initial material structure investigated by CT is shown. The standard configuration of the evaluation is the 2 mm—configuration. Every curve shows the unique specimen number and the varying parameter (temperature or thickness). The full specimen number, initial thickness and compaction parameters are listed in Table A1 in the Appendix A.

### 4.1. Initial Material Structure

**Figure 9:**
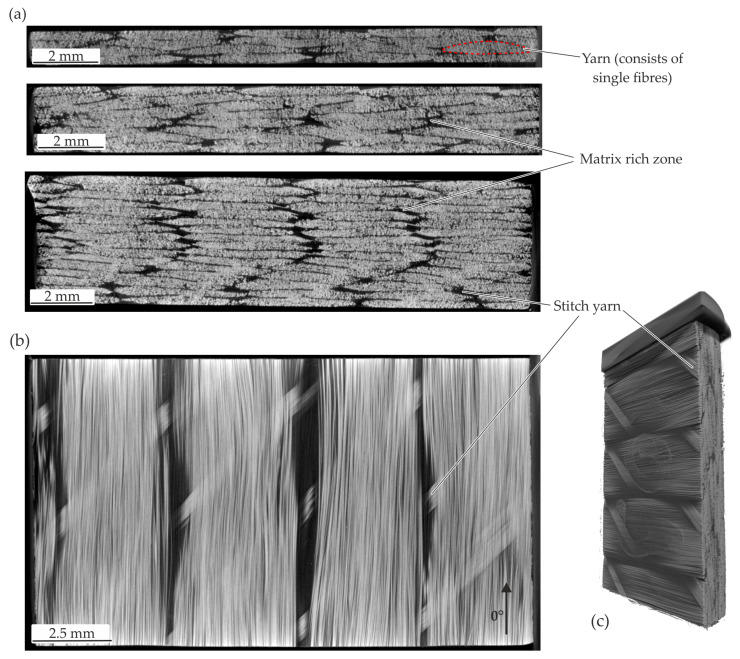
CT analysis of the initial material structure (**a**) cross-section in thickness direction of 1 mm, 2 mm and 4 mm specimens (**b**) cross-section in fibre direction of the 2 mm specimen (**c**) 3D view of the 2 mm specimen.

The initial material structure of the three thickness configurations is investigated and shown in Figure 9. The TPC layout is characterised by UD layered yarns consisting of single fibres. Hence, a resultant non-isotropic material behaviour in accordance to the experimental tensile tests can be assumed. Each yarn is hold in position by an undulated stitch yarn. In all specimens the cross-section of yarn and matrix rich zones beside can be seen (cf. Figure 9a). The yarns of the both 1 mm and 2 mm configuration show nesting effects and approximately similar cross section dimensions of the yarns. While the stacking of the 4 mm specimens shows thinner yarn sections and a sequential stacking with less nesting effects. Therefore, matrix rich zones occur next to each other. In general, the fibre paths of the yarns are in good agreement with the global 0°—direction except in the areas of the stitch yarn where ondulations occur. An impression is given with the 2 mm configuration with a cross section in the middle of the specimen from top view (cf. Figure 9b) and a 3D view (cf. Figure 9c).

### 4.2. Compaction

**Figure 10:**
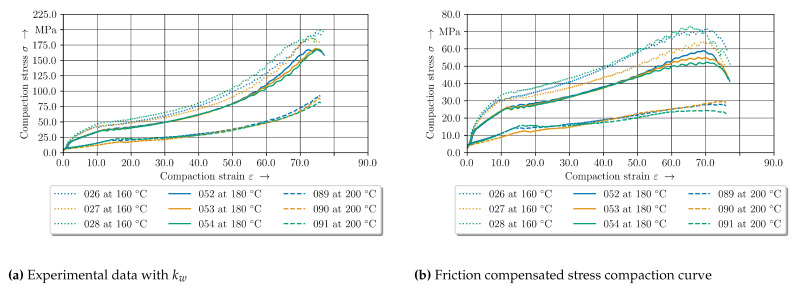
Experimental stress compaction curves of the 2 mm–configuration for the three different temperature levels (**a**) without friction compensation (**b**) with friction compensation according to [43].

The compaction curves of the 2 mm—configuration for the different temperature levels show the temperature-dependent compaction behaviour for the measured specimen in Figure 10. Firstly, the difference between the compensated and non-compensated stress-strain curves can be seen. In Figure 10a it is evident that an increase in compaction increases the in thickness stress. In comparison to the compensated stress-strain curves, with higher compaction levels the influence of the friction increases, whereas the compaction curve increases linearly. A distinction has to be made between the temperature level of 200 °C and the other temperature levels.

The experimental data curves below 200 °C have the same shape in comparison to the clinching curve (cf. Figure 1) except the shape of the offsetting phase. The curves for 160 °C and 180 °C show a higher ascent and more undulation at a compaction strain ϵ of approx. 15%. Especially at higher compaction levels above 50% the curves show a high variation. The difference between these two lower temperature sets are small in comparison to the 200 °C results. In conclusion, the compaction force and the ascent are decreasing strongly with increasing temperature. Moreover, the variation is reduced, which indicates different material behaviour. All curves oscillate between 10% to 20% compaction strain ϵ in common.

### 4.3. Elastic-Plastic Material Behaviour of GF/PA6 under Pressure at 160 °C

**Figure 11:**
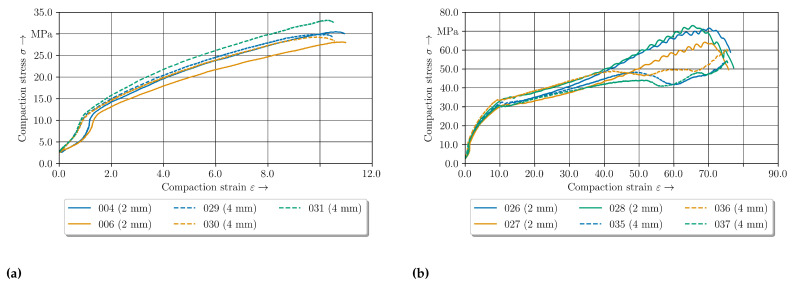
Compaction curves at 160 °C for two configurations. (**a**) 10% compaction level. (**b**) 75% compaction level.

The compaction stress–strain curves of two compaction levels are given in Figure 11. Despite initial pressure of 500 N ( 3.5 MPa) up to 1% compaction, the force increases strongly which can be seen as elastic behavior of the tpc. The curves show a progressive curve profile especially for the 2 mm—configuration. The progressive profile can be explained with setting effects of the material occuring above 500 N. With regard to these setting behaviour the effect decreases with increasing temperature level.

Especially from 2% to 10% strain the curves can be considered to be equal. At a compaction level of approx. 10% to 15% the stress curve oscillates for both configurations. The two data sets begin to differ at approx. 40% (Figure 11b).

The compaction curves for the 2 mm—configuration have more ascent whereas the compaction stress of the 4 mm—configuration remains constant or drop until 65%. Afterwards the stress increases significantly. For the standard 2 mm—configuration the stress-strain curves oscillate with increasing compaction level above 50% and decrease strongly at 70%.

**Figure 12:**
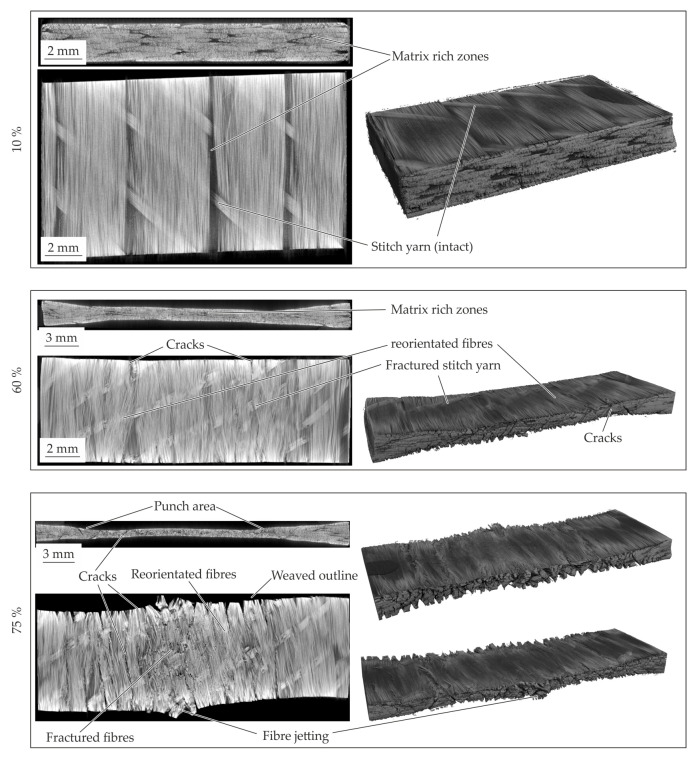
CT analysis of the 2 mm—configuration for the different compaction levels at 160 °C.

The resultant material structure for the different compaction levels of the 2 mm—configuration is presented in Figure 12. At the 10% compaction strain no significant change of the material structure can be seen. The yarn cross section and the stitch yarn within the textile architecture are still intact. An elongation of the specimen in fibre direction can be detected at the outer surface. At a compaction level of 20% the stitch yarns failed and reorientation and setting processes can occur. These result in fibre reorientation while yielding (Figure 12—60%). The yielding processes in transverse direction are characterized by a transverse displacement of the fibres and matrix under compaction load. Since no flow processes of the matrix such as percolation [20] occur, no homogenization effects of the matrix rich zones with the fibres can be seen. This is evident by the observed matrix rich zones despite the high compaction level of 60%. The compaction leads to setting effects of the yarns and fibres driven by shear and normal stresses in transverse direction. These resulting shear and normal deformations lead to inter- and intrafibre failure. These phenomena can be explain the oscillating curves. The evident cracks are initiated at the outer areas and proceed in thickness direction as well as in fibre direction (cf. Figure 12—60%), whereby the ductile matrix can compensate the occurring deformations. This deformation behaviour characterized by setting effects and failures led to the significant variation in the compaction curves (cf. Figure 11b). At the final compaction level of 75% (cf. Figure 12—75%) matrix rich zones still can be detected. The high compression force at this level leads in the center of the specimen to fibre failure and fibre jetting. In the center area no material can yield which limits the deformation under load. Additionally, the compaction force and the Possion’s ratio of the fibres result in a tension stress in fibre direction. When the tension exceeds the fibre strength, fibre failure occurs. After failure, the fibres were pressed out in the fibre direction, which can be described as fibre jetting.

In conclusion, the compaction process at 160 °C is driven by a solid state behavior of the fibres as well as the matrix. In comparison to normal temperature levels the matrix is more ductile and shows no significant failure behavior especially in the matrix rich zones.

### 4.4. Elastic-Plastic Material Behaviour of GF/PA6 under Pressure at 180 °C

**Figure 13:**
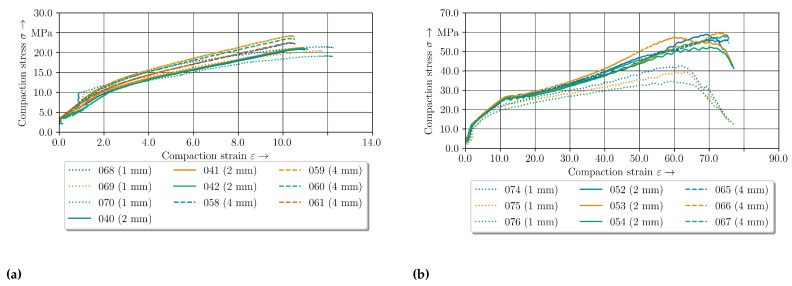
Compaction curves at 180 °C for three configurations. (**a**) 10% compaction level. (**b**) 75% compaction level.

The compaction curves for all three thickness configurations are given in Figure 13. The curves up to 10% compaction strain are similar (Figure 13a). With increasing compaction, the 1 mm—configuration behaves less stiff. In accordance to the 160 °C results, an oscillating of the stress curve is evident around 10% to 20%. Whereat for the 1 mm—configuration the oscillation is reduced in comparison to the other configurations. Especially at 60% resultant compaction stress decreases up to approx. 12 MPa. Both the standard and 4 mm—configuration show the same material behaviour until an oscillating at 50% begins. Additionally, an oscillating of the standard configuration can be observed at a compaction level from 10% to 20% whereas the other configuration shows no oscillating phenomena. The oscillation of the standard configuration curve can be seen until the stress decreases similar to the 1 mm—configuration. Furthermore, the descent looks equal. It can be assumed that the same phenomena during compaction occur in this compaction phase.

The differences in the curves are based on the change of the inner material structure and are investigated by CT-analysis.

#### 4.4.1. Standard 2 mm—Configuration

**Figure 14:**
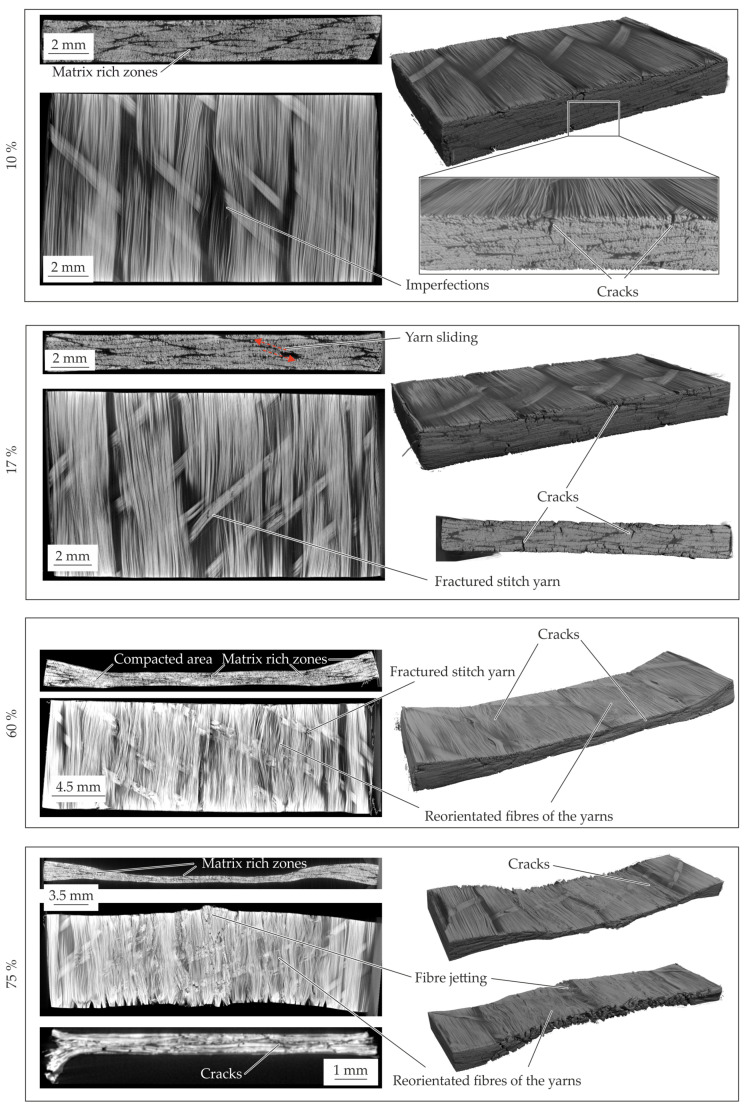
CT analysis of the 2 mm–configuration for the different compaction levels at 180 °C.

The CT analysis of the 2 mm—configuration with an additional compaction level of 17% are shown in Figure 14. The material structure at 10% compaction strain show in comparison to the initial structure no significant changes. The first intrafibre failures in the yarns occur at the fibre endings. Due to the variation of the compaction curves around 10 up to 20% in all configurations, the inner material structure is investigated at 17%. At this level all occurring phenomena can be seen (cf. Figure 14—17%). The intrafibre cracks increasing and matrix rich zone are still visible. One of the major phenomena is the failure of the stitch yarn. It can be concluded that the stitch yarns were elongated under compaction force while the yarns sliding and shifting.

When the critical strain of stitch yarn is achieved this yarn failed which leads to a reduction of the compaction stress. This process is repeated until all stitch yarns failed. Thereby, the oscillation of the stress-strain curve around 10% to 20% can be explained (cf. Figure 13b). The failure of the stitch yarns enable a reorientation of the yarns which can be seen in the cross-section figure. Further compaction leads to a squeeze flow transverse to the fibres and more stitch yarn failures (cf. Figure 14—60%).

At the last compaction level of 75% intra- and interfibre failure occurs. The cracks propagate in thickness and longitudinal direction through the whole specimen. In the cross-section along the fibres it can be seen that the fibres are just reoriented in-plane. Due to the fibre failure by compression fibre jetting can be observed as well (cf. Figure 14—75%). The cracks in the specimen leading to a reduced resistance against compaction is shown by the drop at 75%.

#### 4.4.2. 1 mm—Configuration

**Figure 15:**
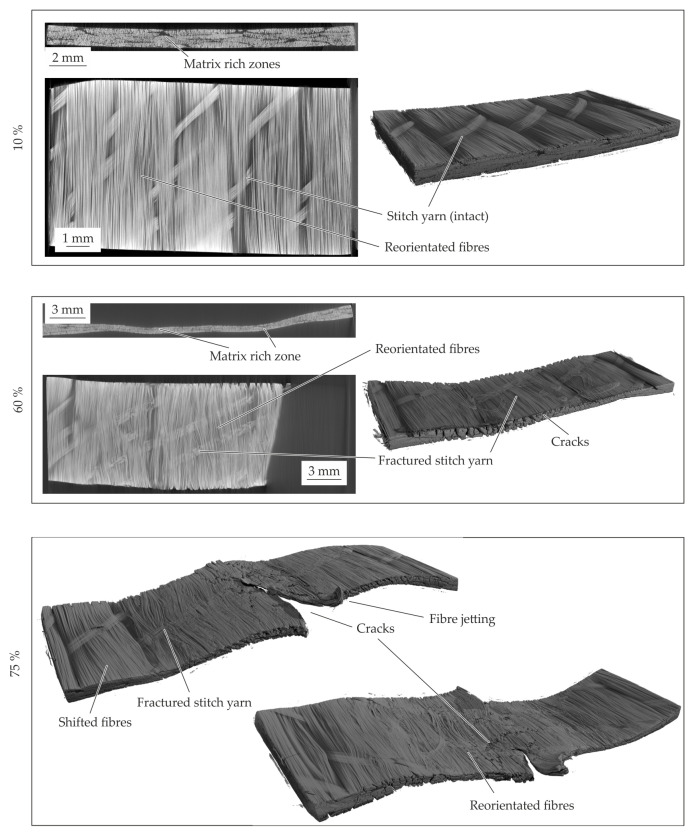
CT analysis of the 1 mm—configuration for the different compaction levels at 180 °C.

The resultant material structure of the specimen with a thickness of 1 mm, compacted to a defined compaction level is given in Figure 15. In comparison to the initial material structure (cf. Figure 9) no voids or fibre failures can be detected at a compaction level of 10%. Moreover, the deformation phenomena in comparison to the standard configuration are similar. A fibre shifting on the left side is evident and an in-plane shear deformation can be concluded. Caused by the thin initial thickness, this effect can clearly be seen in comparison to the other configurations. According to the standard configuration the stitch yarn failed at 10%, which can be seen at the singularity (cf. Figure 13a). At a compaction level of 60% fractures according to the compaction levels of 75% of the standard configuration occur. Furthermore, a fibre reorientation beneath the punch area can be seen at the surface and in the top view of the mid plane. On the edges of these areas interfibre failure and intrafibre failure (cracks) occur. With further compaction up to 75% the cracks in the punch area grow until the cracks split the specimen. The reorientation of the fibres increases, whereas the fibres next to the punch where shifted caused by the squeeze flow. Due to the squeeze flow cracks in terms of interfibre failure and intrafibre failure in thickness direction and in along the fibres occur. The CT analysis indicates a crack initiation at the lateral surface of the fibre endings. When the crack runs along the whole specimen, the compaction stress decreases significantly at approx. 65% (cf. Figure 13b).

In conclusion, the squeeze flow process caused by compaction leads to a shifting of fibres in transverse direction. The inner fibres under the punch and also the fibres on the surface were reoriented at in-plane direction. Due to the initial thickness, the failure phenomena can be observed at lower compaction levels in comparison to the standard configuration.

#### 4.4.3. 4 mm—Configuration

**Figure 16:**
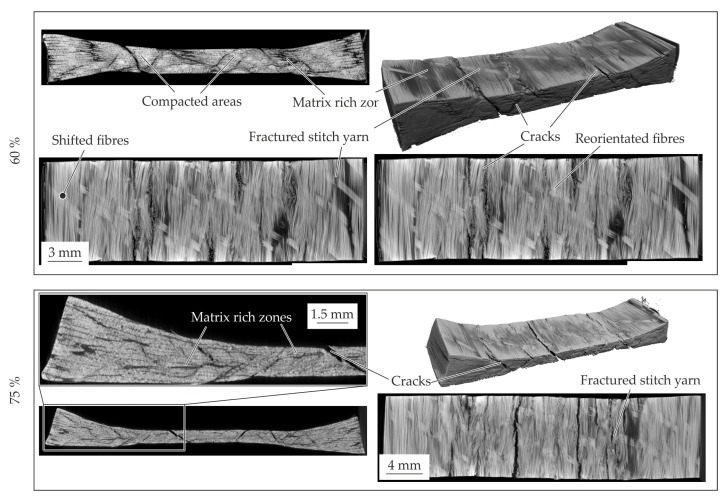
CT analysis of the 4 mm—configuration for the different compaction levels at 180 °C.

The inner material structure of the 4 mm—configuration during compaction can be seen in Figure 16. In comparison to the standard configuration, the 10% compacted material show an intact tpc and no differences. The matrix rich zones are similar to the initial configuration. In contrast to the standard configuration, a bulge on left and right side of the specimen is formed due to the compaction. Under a compaction strain of 60% the material structure shows a crack pattern (cf. Figure 16—60%). The crack angles in thickness direction seems similar to each other and can be seen in the area under the punch and at squeezed material beside. It can be described by sliding angles. The phenomena and the crack growth increasing with the further increased compaction level. During these crack propagation also the fibre reorientation at in-plane direction can be seen. Due to the sliding effects and the crack propagation the equal stresses in comparison to the other standard configuration can be explained (cf. Figure 13). Since the 4 mm—configuration has more material in thickness direction in the squeeze flow area, the sliding phenomena occur also for higher compaction strains. This leads to higher compaction stress at a compaction level of 75% in comparison to the other configurations.

### 4.5. Elastic-Plastic Material Behaviour of GF/PA6 under Pressure at 200 °C

**Figure 17:**
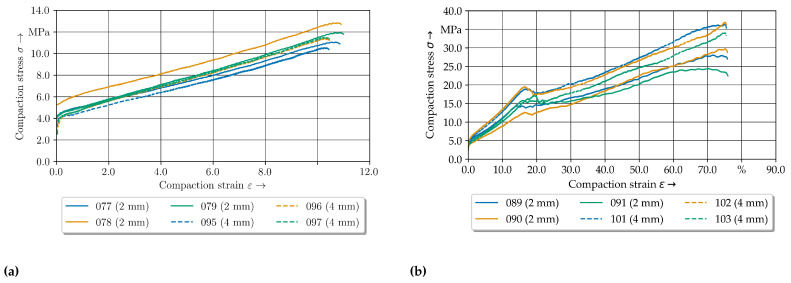
Compaction curves at 200 °C with the two tested configurations. (**a**) 10% compaction level. (**b**) 75% compaction level.

The temperature level of 200 °C is next to the Vicat temperature of PA6. The resultant compaction curves are given in Figure 17. It can be seen that the force level of both configurations are in the same amount of magnitude until 60% compaction strain. Therefore, the 2 mm—configuration is investigated by CT analysis.

**Figure 18:**
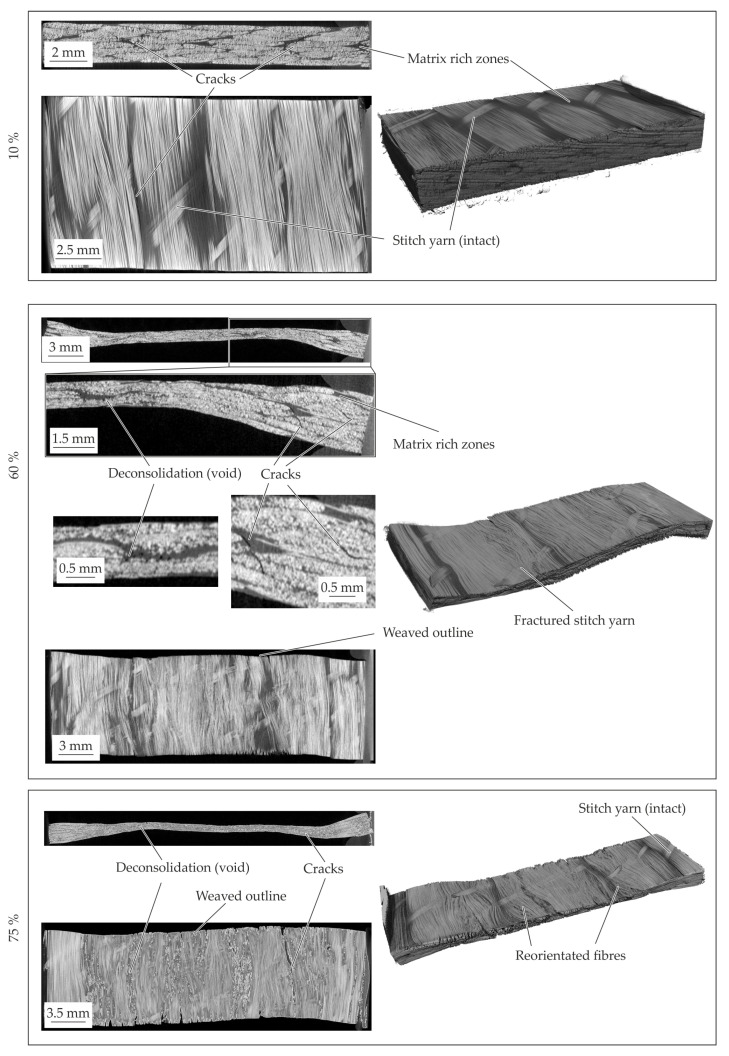
CT analysis of the 2 mm—configuration for the different compaction levels at 200 °C.

The material structure of the different compaction levels are given in Figure 18. In accordance to the results of 180 °C also intrafibre cracks but also cracks in the matrix rich zones occur at a compaction level of 10%. The stitch yarn is also still intact. With increasing compaction level up to 60%, voids can be seen. The voids can be caused by vaporisation of residual humidity within the matrix or vacuoles due to shrinkage effects and recrystallisation within the cooling step. This could also be the reason of the cracks in the matrix rich zones and between the fibres. Additionally, at this compaction strain, the stitch yarn also failed. This enables the reorientation of the fibres in the punch area. Due to the softened matrix also the fibres on the sides are shifted and reorientated while pressing. This stands in contrast to the other two temperature levels. It also can be seen that voids inside the specimen increase.

## 5. Discussion

The analysis of the inner material structure at different compaction levels shows compressing, sliding and squeeze flow phenomena. At higher compaction levels crack occur. The phenomena at the fibre level resulting in fibre reorientation via shifting and bending. It can be seen that the compaction behaviour strongly depends on the temperature resulting in an increasing ductility of the matrix. Due to these softening of the matrix the phenomena such as yarn shifting, bending, compaction and sliding occur more easily. Furthermore, the textile architecture has to be taken in to account. In all compaction curves between 10 up to 20% compaction oscillating phenomena can be seen. By using CT analysis it can be concluded that the failure of the stitch yarns lead to these effects. The failed stitch yarn allows a new reorientation of the yarns in terms of sliding and shifting under compression stress. Thus, the textile architecture is essential for the compression behavior while forming and the required compaction force especially at higher deformations.

It can be noticed that next to the Vicat temperature around 200 °C crack initiation in both the matrix rich zones and yarns occur which stand in contrast to the temperatures below. This cracks can be initiated by deconsolidation of moisture evaporation. Those phenomena often can be observed at heating processes up to melting temperature [46]. The cracks caused by compaction can be seen in all compaction levels above 10% and can occur between the sliding yarns especially in areas with less matrix. These areas cannot compensate the shear stresses which lead to intra-fibre failures. These sliding effects requires a special amount of yarns and matrix in the pressing area. When the initial textile architecture of yarns is eliminated and just the fibre bundles are compacted, the maximum compaction level with material structure change is achieved. After fibre failure and crack growth through the whole specimen, the material resistance drops immediately.

**Figure 19:**
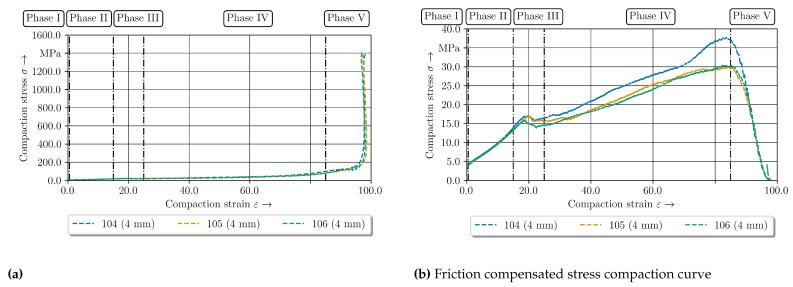
Experimental stress compaction curves of the 2 mm—configuration for 200 °C up to a compaction force of 200 kN (**a**) without friction compensation (**b**) with friction compensation according to [43].

For identification of these phenomena it is crucial to compensate the friction in the compaction stress strain curves [43]. By using the raw experimental data, the compression force and therefore the compression stress increases significantly. This was evaluated by a force controlled compaction test with a maximum force of 200 kN for the 4 mm—configuration at 180 °C. The compaction curve can be seen for both raw and friction compensated data in Figure 19.

In conclusion of the investigations, the compaction curve can be classified into five phases shown in Figure 19b in contrast to the four phases of the clinching process. The first compaction phase is elastic and will become inelastic without damage of stitch yarn (up to 0.5%). In the second phase (up to 15%) a structure deformation by reorientation of yarns occurs. After the inelastic compaction the reorientation process occurs. These processes proceed with less stiffness in comparison with the first phase. Due to the limited elongation of the stitch yarns these types of structure deformation are also limited and leading to an increasing compaction stress.

Hence, third phase is defined by the stitch yarn failure resulting in an oscillating stress curve (15% to 25%). The unbounded yarns can slide until the next stitch yarn stops this process. So the stress increases up to the failure of the stitch yarn. This phenomenon and oscillation of the stress strain curve are repeated until all preventing stitch yarn failed. Afterwards phase four with crack initiation and crack growth based on intra- and interfibre failure occurs (up to 92%). The cracks are initiated by yarn sliding during the squeeze flow. Depending on the initial thickness these phenomena are also limited. The limit is achieved at phase four when sliding is no longer possible. The yarn including matrix is compacted to the most efficient package density. The following phase five is a flow pressing of fibre until the material resistance drop caused by crack growth along the fibres through the whole specimen. The measured stress increases caused by friction phenomena (Figure 19a). These experimental results create accurate input data for describing the material behaviour of the flow pressing phase in a numerical simulation. This enables a detailed description of the resultant undercut and material structure. Thereby the predictions of the load bearing behavior are assessed. In respect to the determined friction coefficient at 160 °C and considering the three different types of friction, the compensation of the friction has to be carried out with FC for each temperature level to increase the accuracy. Comparing the determined constant FC and the experimental data forTwintex/Polypropylene of [35], the friction is in the same amount of magnitude for the steady case. It can be assumed, that the presented evaluation method is capable for determination of FC.

## 6. Summary

The present paper focuses on the flow pressing phase of a thermally assisted clinching process of metal and TPC. Therefore an experimental setup is developed to generate a squeeze flow according to the clinching technology. The compaction behaviour is described with compaction stress- strain curves. A detailed investigation of the material structure is made with CT-analysis. This approach enables a deeper knowledge of reorientation phenomena and occurring failure of the TPC while compaction. The compaction levels are chosen accordingly to the clinching process whereas other paper focus on compaction while forming. The results show a significant influence of the temperature and the textile architecture to the compaction stress- strain curves. The occurring phenomena are sliding and reorientation effects in terms of fibre shifting and bending. This phenomena resulting in intra-fibre failure cracks. When the process temperature is up to the Vicat temperature, deconsolidation can be seen in contrast to the temperatures below. Furthermore, matrix cracks can occur. It has been shown, that the experimental determined compaction stresses has to be compensated for the existing friction conditions to relate compaction phenomena of the material structure to the required forming stresses. Further work should focus on the implementation into numerical simulations. Additionally, due to the hydrostatic stress state in the die, investigations of the hydrostatic material behaviour and tool-ply interaction should be conducted. Furthermore the behaviour at higher temperatures should be taken into account, to gain a deeper knowledge for other joining processes.

## Figures and Tables

**Table 1:** Specification of the used material.

TPC Sheet	
Material	GF/PA6
Configuration	UD
Fibre volume content	44%
Thickness *d*	1 mm, 2 mm, 4 mm
Specimen area	9 × 16 ${\mathrm{mm}}^{2}$

**Table 2:** Test specification for compaction specimens with the different thicknesses.

*d*	*T*	Compaction Level in %					
in mm	in °C	10	17	40	60	70	75
1	180	x			x		x
2	160	x		x	x	x	x
	180	x	x	x	x	x	x
	200	x		x	x	x	x
4	160	x			x	x	x
	180	x			x	x	x
	200	x			x		x

**Table 3:** Test specification for compaction specimens for CT.

Parameter	Unit	Value
Acceleration voltage	kV	80
Tube current	μA	200
Exposure time	ms	1000
X-ray projections		1440 (4 per 1°)
Source object distance	mm	90
Source image distance	mm	1200
Voxel size	μm	15.0
Filter	mm	0.2 (copper)

## Data Availability

Not applicable.

## Abbreviations

The following abbreviations are used in this manuscript:

**2D**	2-dimensional
**3D**	3-dimensional
**FC**	friction coefficient
**FVC**	fibre volume content
**GF**	glass fibre
**CF**	carbon fibre
**GFRP**	glass-fibre-reinforced
**CFRP**	carbon-fibre-reinforced
**PA6**	polyamide 6
**PEEK**	polyetheretherketone
**PEKK**	polyetherketoneketone
**PPS**	polyphenylensulfide
**PP**	Polypropylene
**TPC**	thermoplastic composites
**UD**	unidirectional

## Appendix A. Specification of the Specimens

**Table A1:** Process parameters and initial thickness for every specimen number.

Specimen Name	Initial Thickness in mm	Compaction Level in %	Temperature *T* in °C
A03_F_004	2.11	10	160
A03_F_006	2.11		
A03_F_026	2.27	75	160
A03_F_027	2.05		
A03_F_028	2.09		
A03_F_029	4.22	10	160
A03_F_030	4.21		
A03_F_031	4.18		
A03_F_035	4.18	75	160
A03_F_036	4.22		
A03_F_037	4.19		
A03_F_040	2.21	10	180
A03_F_041	2.18		
A03_F_042	2.12		
A03_F_052	2.07	75	180
A03_F_053	2.06		
A03_F_054	2.03		
A03_F_058	4.32	10	180
A03_F_059	4.20		
A03_F_060	4.19		
A03_F_061	4.23		
A03_F_065	4.19	75	180
A03_F_066	4.24		
A03_F_067	4.19		
A03_F_068	1.06	10	180
A03_F_069	1.06		
A03_F_070	1.05		
A03_F_074	1.05	75	180
A03_F_075	1.05		
A03_F_076	1.06		
A03_F_077	2.21	10	200
A03_F_078	2.01		
A03_F_079	2.04		
A03_F_089	2.14	75	200
A03_F_090	2.34		
A03_F_091	2.06		
A03_F_095	4.19	10	200
A03_F_096	4.11		
A03_F_097	4.10		
A03_F_101	4.09	75	200
A03_F_102	4.08		
A03_F_103	4.21		
A03_F_104	4.17	200 kN	200
A03_F_105	4.17		
A03_F_106	4.20		
